# Impact of the Chicago Earned Income Tax Periodic Payment intervention on food security

**DOI:** 10.1016/j.pmedr.2019.100993

**Published:** 2019-11-06

**Authors:** Flavia Cristina Drumond Andrade, Karen Z. Kramer, Andrew Greenlee, Adam Nephi Williams, Ruby Mendenhall

**Affiliations:** a School of Social Work, University of Illinois at Urbana-Champaign, USA; b Department of Human Development and Family Studies, University of Illinois at Urbana-Champaign, USA; c Department of Urban and Regional Planning, University of Illinois at Urbana-Champaign, USA; d Departments of Sociology and African American Studies, University of Illinois at Urbana-Champaign, USA

**Keywords:** Food insecurity, Earned Income Tax Credit, Intervention, Periodic payment

## Abstract

This article examines the Earned Income Tax Credit Periodic Payment Pilot and its effectiveness in reducing food insecurity for low-income households. Low-income families in Chicago who were eligible for the Earned Income Tax Credit provided data over four waves of data collection between 2014 and 2015. We utilize longitudinal random effects logit models to test the likelihood of experiencing food insecurity. The sample was composed mostly by women with low educational levels. The intervention significantly decreased the likelihood of experiencing food insecurity over time (T2: β = −0.23, p = .581; T3: β = −0.89, p < .10; T4: β = −2.21, p < .01). The Periodic Payment Pilot seems effective at reducing food insecurity in low-income families. Further research should examine how changes to the Earned Income Tax Credit payment distribution could improve the lives of low-income families, specifically concerning food insecurity.

## 1 Introduction

Food insecurity (FI) is a major public health issue in the United States. In 2017, FI affected 11.8 percent of households in the United States, but rates are higher among low-income households, households with young children, households headed by women, and households in urban areas (Coleman-Jensen et al., 2018). The cumulative cost of food insecurity on health care expenditures is estimated at $77.5 billion annually (Berkowitz et al., 2018).

The Earned Income Tax Credit (EITC) is a government assistance program that has been found to increase income and reduce FI (Rehkopf et al., 2014, Schmidt et al., 2016). There is evidence that EITC recipients have improved health behaviors, better birth and child health outcomes and better maternal mental health (Markowitz et al., 2017, Siefert et al., 2004, Spencer and Komro, 2017, Wagenaar et al., 2019). The EITC is paid as a lump sum as part of the annual tax refund, but its annual distribution can be a shortcoming. EITC recipients frequently make use of the lump-sum payment early in the year, but experience financial and budgeting instability throughout the year (Mendenhall et al., 2014, Smeeding et al., 2000). The inability to cover financial emergencies, such as unexpected health bills, fixing a broken car, or attending to unexpected home repairs may result in increased household financial stress that can negatively impact household food security and wellbeing (Mendenhall et al., 2014). Between 1978 and 2010, individuals could opt for either receiving the lump sum payments or to receive a portion in advance through their paycheck, but only 3 percent of eligible individuals utilized the advance payment system (Romich and Weisner, 2000). There were administrative difficulties besides small amounts of disbursements that made the advanced payments not appealing to employers and recipients (Holt and Solutions, 2008).

In this study, we evaluate a modified version of the advanced periodic EITC payments. The EITC Periodic Payment Pilot was an intervention program designed to relieve economic stress for low-income families throughout the year by providing up to 50 percent of the expected EITC payment in advance instalments. EITC recipients use most funds to pay debt or spend it on basic consumption, such as food (Despard et al., 2015). Nonetheless, low-income households are more likely to experience FI and also less likely to have social networks that can provide food or groceries at difficult financial times (Gundersen and Ziliak, 2014). Therefore, we expect periodic EITC payments to protect families from FI, which often occurs when families reduce the only flexible expense they have – their food budget (Bruening et al., 2012) by providing a steady financial buffer throughout the year. Specifically, in this study, we compare the effects of receiving the EITC as a lump sum to receiving up to 50 percent of the annual EITC payment as a series of quarterly periodic payments on food security.

## 2 Materials and methods

### 2.1 Sample

We utilize data from the Chicago Earned Income Tax Credit Periodic Payment Pilot that was conducted in 2014–2015. In an effort to lower financial stress, participants in the intervention group (n = 343) received four periodic payments totalling 50 percent of their 2014 projected tax credit, but up to $2000. Participants in the control group (n = 164) received the traditional lump sum EITC payment in conjunction with their annual tax refund. Surveys were completed online and/or by phone, in four waves. Baseline data (T1) were collected, in March and June of 2014, before any advanced periodic payment was made to the intervention group and around the time participants received their tax refund and EITC for the 2013 tax year. The second wave of surveys (T2) occurred in June and July, after the first two periodic payments were received by the intervention group. Following receipt of the third payment in October, the third wave (T3) was carried out in November and December. The last periodic payment was received in December 2014. The final surveys (T4) occurred between January and May 2015 after the fourth and last periodic payment was made. Participants in both arms of the study filed their 2014 tax returns between January 1st and April 15, 2015. Individuals in the control group received the traditional EITC lump sum during 2015, after mid-February. Therefore, the control group received the tax return at the end of the study. Individuals in the treatment group received the other half of their credit during 2015 when they returned to the Center for Economic Progress to file their 2014 taxes. Details about the data collection and study design have been published elsewhere (Andrade et al., 2017).

Of the participants in the sample, 64 had missing data on selected variables (i.e. food insecurity, comfort with income level, and public assistance) in the baseline, which results in a total of 443. In the T4, there were 278 participants, 68 in the control group and 210 in the intervention group.

### 2.2 Measures

#### 2.2.1 Food insecurity

FI was measured as a categorical variable: any FI (=1) versus food security (=0). Any FI was defined as a respondent answering that they did not have enough money to feed their family at least one day during the last month.

#### 2.2.2 Comfort with income level

Respondents were asked how comfortably their family can live on their total household income. This is measured on a 4-point scale, from not at all comfortably to very comfortably.

#### 2.2.3 Public assistance

Participants were asked if they had received public assistance related to supplemental nutrition assistance program (SNAP), temporary assistance for needy families (TANF) and supplemental security income (SSI). We created a dummy variable to indicate whether they had received any of these benefits.

#### 2.2.4 Financial stress

Perceived financial stress was measured using the 8-item InCharge Financial Distress/Financial Well-Being scale (IFDFW) (Garman and Sorhaindo, 2005). Responses were made using a 1 to 5 scale where 1 is “strongly disagree” and 5 is “strongly agree.” A sample item is: “I am confident that I can find the money to pay for a financial emergency that costs about $1000.” Alpha reliability for the 8-item scale was 0.82.

#### 2.2.5 Covariates

Covariates included, sex, educational attainment [less than high school, high school (HS) or more, not reported], number of people in the household, and number of children in the household.

### 2.3 Statistical analysis

Random-effects logit models were used to analyze the effects of the EITC intervention on the likelihood of FI over time. First, we measured the effects of the EITC intervention on FI, controlling for demographic and household characteristics. Second, we added the time variable and the interaction term between time and intervention variable as a way to test the interaction of the intervention over time to estimate their effect on FI. All data analyses were conducted using statistical software STATA SE 14.

## 3 Results

The majority of the sample was composed of low-educated women (see Table 1). Those in the intervention group had a significantly greater number of people living in their household (*M* = 3.37, *SD* = 1.50) than the control group (*M* = 2.56, *SD* = 1.50). There was also a significant difference in the number of children, with a greater mean among those in the intervention group. The control group had a lower percentage of food insecure households at T1 (31.97%) than the intervention group (36.82%), though this difference was not statistically significant. The intervention and control groups also did not differ at T1 on how comfortably participants could live on their income, or on their financial stress. A higher proportion of participants in the intervention group (82.09%) reported receiving public assistance than the control group (66.67%).

**Table 1:** Descriptive Statistics of the EITC study at baseline, Chicago: 2014–2015 (n = 443).

Variables	Control Group	Intervention Group	p-values
Food Security (%, n)			
Yes	31.97 (47)	36.82 (109)	0.314
No	68.03 (100)	63.18 (187)	
Respondent Gender (%)			
Female	84.35 (124)	93.24 (276)	<0.001
Male	14.97 (22)	2.36 (7)	
Other	0.68 (1)	4.39 (13)	
Education (%, *n*)			0.902
Less than HS	55.78 (82)	57.43 (170)	
HS or more	36.05 (53)	35.47 (105)	
Not reported	8.16 (12)	7.09 (21)	
Mean Number of Children (*SD*)	1.52 (1.07)	2.53 (1.35)	<0.001
Mean Household Size (*SD*)	2.56 (1.50)	3.37 (1.50)	<0.001
Mean Comfort with Income (*SD*)	2.02 (0.81)	2.04 (0.69)	0.8204
Receive Public Assistance (*%, n*)			<0.001
Yes	66.67 (98)	82.09 (243)	
No	33.33 (49)	17.91 (53)	
Mean Financial Stress (SD)	3.50 (0.80)	3.64 (0.74)	0.0645

**Fig. 1:**
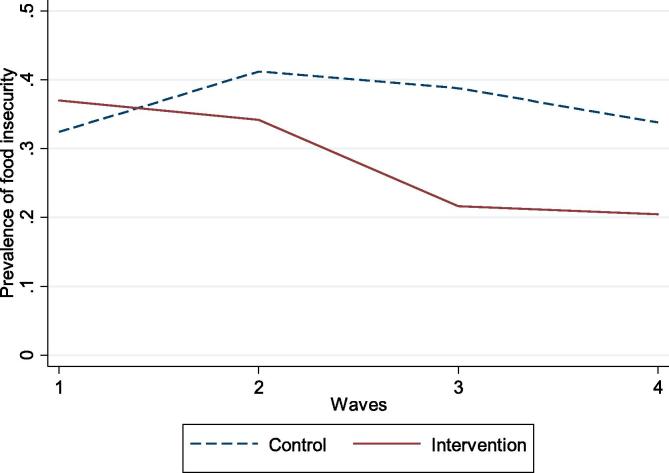
Prevalence of food insecurity in the Earned Income Tax Credit Intervention over Time, Chicago: 2014–2015.

Fig. 1 shows the prevalence of food insecurity during the study period. Prevalence was similar at baseline, but whereas the control group seems to have increased in wave 2 and only slightly decreased at wave 4, the intervention group observed declines in the prevalence over time, with lower prevalence rates particularly at T3 and T4.

Results from the random-effects logit model showed respondents’ comfort with income level was significantly linked to a lower probability of FI (β = −0.41, p < .05) and their level of financial stress was significantly linked to a higher probability of FI (β = 0.96, p < .001), while all other control variables were not statistically significant (Table 2, Model 1). Including the variable for time and interaction with group assignment (Model 2) indicated that there was no significant difference in the likelihood of experiencing FI over time compared to T1 (T2: β = 0.19, p = .588; T3: β = 0.11, p = .780; T4: β = 0.83, p = .184) or between the control and intervention groups (β = 0.22, p = .507). Interacting intervention group status with time (Model 2) showed that the intervention did significantly decrease the likelihood of experiencing FI over time (T2: β = −0.23, p = .581; T3: β = -0.89, p < .10; T4: β = −2.21, p < .01).

**Table 2:** Estimated Parameters (SE) from Random-effects Logit Models of Earned Income Tax Credit Intervention on the Likelihood of Food Insecurity over Time, Chicago: 2014–2015.

	Model 1			Model 2		
	β	SE	p-value	β	SE	p-value
Intervention Group (Ref: Control)	−0.20	0.24	0.422	0.22	0.32	0.507
Time (Ref: Time 1)						
Time 2				0.19	0.36	0.588
Time 3				0.11	0.41	0.780
Time 4				0.83	0.63	0.184
Time*Intervention Group						
Time 2				−0.23	0.42	0.581
Time 3				−0.89	0.47	0.057
Time 4				−2.21	0.77	0.004
Respondent Gender (Ref: Female)						
Male	−0.02	0.45	0.965	−0.03	0.46	0.950
Other	0.07	0.54	0.899	0.09	0.56	0.873
Education (Ref: Less than HS)						
HS or more	0.12	0.22	0.586	0.13	0.23	0.567
Not Reported	−0.01	0.36	0.975	0.00	0.38	0.998
Number of children	−0.15	0.16	0.365	−0.15	0.17	0.387
Household Size	0.18	0.14	0.198	0.18	0.14	0.205
Comfort with Income	−0.41	0.12	0.001	−0.32	0.13	0.016
Receive public assistance (Ref: No)	−0.13	0.21	0.526	−0.19	0.21	0.366
Financial Stress	0.96	0.13	<0.001	0.95	0.14	<0.001
Constant	−3.76	0.69	<0.001	−3.99	0.73	<0.001

Significance *p < .05 **p < .01 ***p < .001.

## 4 Discussion

The purpose of this article was to evaluate whether receiving a portion of the EITC as advance payments resulted in greater food security for low-income families. We found that compared to a control group that received a traditional lump sum payment, periodic payments seem to be associated with lower levels of FI at T3 and T4. Rehkopf et al. (2014) found that FI decreased after receiving the EITC lump sum. In our study both groups had received the lump sum at baseline, so results point to no differences initially (T1 and T2), but FI seems to decrease for the intervention group later on.

Cash and food assistance programs seem to reduce FI in low-income families (Schmidt et al., 2016), but there is further room for changes to current programs that can galvanize these benefits. This study indicates that offering an EITC advanced periodic payment option could improve the food security of low-income families throughout the year. This conclusion is supported by research which shows that advanced periodic EITC payments reduce perceived financial stress (Kramer et al., 2019), which is positively associated with depressive symptoms (Andrade et al., 2017). This is important because parents’ and children’s physical and mental health are associated with household food security (Anderson et al., 2016, Gundersen and Ziliak, 2014, Liu et al., 2014).

Our study underlines the importance of considering how policy changes could affect the well-being of families in the United States, but there are some limitations that must be noted. The foremost limitation is our measure of FI, which is self-reported as not having enough food to feed one’s family for at least one day during the previous month, which is not in accordance with the USDA definition based on a 18-item scale (Radimer, 2002). Nonetheless, the measure from our study is similar to an item from the USDA six-item short form, which classifies households as food-insecure if participants responded affirmatively to at least two of the six items (Furness et al., 2004). However, there is evidence that single-item measures underestimate FI (McKechnie et al., 2018). Another limitation of our study is our small sample and the non-randomization of control and intervention groups. Since the participants self-selected themselves into the intervention group, we are unable to generalize our findings to the general population. However, since any future EITC periodic payment option is likely to be voluntary and complementary to the existing lump sum payment, the self-selection of individuals into these groups likely reflects the preferences of individuals, which is important information for program design and evaluation. We believe that the value of this additional information outweighs the potential limitations associated with non-random group selection. In addition, we are unable to control for the amount of EITC received as we have limited information for the control group. Even though we control for financial stress, which is a subjective measure on how families perceive their financial status, we were unable to account for it, as well as debts and money borrowed (Kramer et al., 2019). Finally, the sample is composed of residents in the city of Chicago, which limits its generalizability.

In conclusion, we analyzed data from an EITC intervention program, which indicates that advanced periodic payments have the potential to alleviate FI throughout the year for poor families in the United States.

## Funding

This study was funded by the United States Department of Agriculture (second author; Hatch project number ILLU-793-365), by the Chicago Housing Authority and the City of Chicago Office of the Mayor, and the UIUC Research Board (first three authors and last author).

## Ethical approval

All procedures performed in studies involving human participants were in accordance with the ethical standards of the institutional and/or national research committee and with the 1964 Helsinki declaration and its later amendments or comparable ethical standards.

## Informed consent

Informed consent was obtained from all individual participants included in the study.
